# Around the Hospitals

**Published:** 1892-06-04

**Authors:** 


### Around the Hospitals.

**The Royal London Ophthalmic Hospital.**—This is the oldest ophthalmic hospital in the country. It has an international reputation, and is truly national from the fact that patients are sent to it from all parts of the country, as well as from London. We give a report elsewhere of a meeting at the Mansion House last week at which it was resolved to raise £35,000 in order to rebuild the hospital. It is to be regretted that the income during 1891 shows a decrease of nearly £1,000 as compared with the previous year, chiefly owing to the serious falling off in legacies. The fact that Mr. Keith D. Young has been selected to prepare the plans ot the new building should give confidence to the public and secure the immediate subscription of the £35,000. The Committee would be well advised to institute a system of payment by those patients who are willing and able to give something towards the cost of their treatment at the institution.

**Worcester General Infirmary.**—The 147th report, being that for 1891, testifies to the value and importance of this great county hospital. It having come to the knowledge of a few leading men in the county of Worcester that the Infirmary would have to sell out funded property to defray current expenses, they have immediately subscribed £1,284 to obviate so direful a necessity. This action is in striking contrast to the inertness and indifference of the people of the city and county of Oxford in regard to the Radcliffe Infirmary. The cost per bed has been reduced from £42 to £40 in round numbers. We note with pleasure that it was determined on October 19th last to affiliate with the Royal National Pension Fund for Nurses, and that the House Committee has received authority to expend a sum not exceeding £30 in paying premiums on policies to be taken out by the Infirmary for the benefit of its nurses.

**The Queen's Hospital, Birmingham.**—This institution which was made free on January 1st, 1876, issues an excellent annual report full of information, presented in a clear and concise manner. It is in its way a model of what such documents should be. The average residence of each in-patient was 21 days, and the average cost £3 1s. 3d. during 1891, as compared with £2 6s. 7d. in 1886. The income has increased from £6,286 in 1881 to £9,214 in 1891, and the Committee attribute this success in great measure to the loyal co-operation of their official staff, and especially to the services of Mr. Hulme, the general superintendent and Mrs. Willett, the housekeeper, who have held their respective offices for nearly the whole ten years, and have displayed a zeal and fidelity which justly deserve this public testimony. The feature of the year has been the establishment of a nursing institution, which is served by a staff of six nurses trained in the hospital, and which was opened on October 1st, 1891. The results so far attained are amazingly satisfactory, and this branch will no doubt speedily assume great importance and render material service to many private families in Birmingham and the district.

The Quarterly General Court of Governors of the **Middlesex Hospital** was held on the 26th ult. Mr. H. N. Custance occupied the chair, and there was a large attendance, among others being present the Lord Muncaster, Mr. Elliott Lees, M.P., Mr. Noel, C.B., Mr. Bell Sedgwick, J.P., and Mr. Hulke, F.R.S. The Secretary, in reviewing the transactions of the Weekly Board of the hospital since the last Court, stated that the Board had felt great satisfaction at the employment of two of their officers in the important public work of making investigations at the instance of the Royal Commission on Vaccination into an outbreak of small-pox at a large provincial town. The Secretary also reported that the Board had felt it their duty to postpone the execution of several necessary alterations and improvements until next year, in consequence of the closure of the University and Charing Cross Hospitals from unavoidable causes, having regard to the large amount of sickness in the district which would have been left totally unprovided for had the Middlesex Hospital also closed. Donations of £500 from the Goldsmiths' Company, £700 from Baron de Hirsch, £100 from Miss M. A. Leicester were announced, together with a legacy of £2,000 from the late Mr. David Price.

